# Pharmacological BACE1 and BACE2 inhibition induces hair depigmentation by inhibiting PMEL17 processing in mice

**DOI:** 10.1038/srep21917

**Published:** 2016-02-25

**Authors:** Derya R. Shimshek, Laura H. Jacobson, Carine Kolly, Natasa Zamurovic, Kamal Kumar Balavenkatraman, Laurent Morawiec, Robert Kreutzer, Juliane Schelle, Mathias Jucker, Barbara Bertschi, Diethilde Theil, Annabelle Heier, Karine Bigot, Karen Beltz, Rainer Machauer, Irena Brzak, Ludovic Perrot, Ulf Neumann

**Affiliations:** 1 Neuroscience, Novartis Institutes for BioMedical Research, 4002 Basel, Switzerland; 2 Preclinical Safety, Novartis Institutes for BioMedical Research, 4002 Basel, Switzerland; 3 Metabolism and Pharmacokinetics, Novartis Institutes for BioMedical Research, 4002 Basel, Switzerland; 4 Global Discovery Chemistry, Novartis Institutes for BioMedical Research, 4002 Basel, Switzerland; 5 Department of Cellular Neurology, Hertie-Institute for Clinical Brain Research, University of Tübingen and German Center for Neurodegenerative Diseases (DZNE), 72076 Tübingen, Germany

## Abstract

Melanocytes of the hair follicle produce melanin and are essential in determining the differences in hair color. Pigment cell-specific MELanocyte Protein (PMEL17) plays a crucial role in melanogenesis. One of the critical steps is the amyloid-like functional oligomerization of PMEL17. Beta Site APP Cleaving Enzyme-2 (BACE2) and γ-secretase have been shown to be key players in generating the proteolytic fragments of PMEL17. The β-secretase (BACE1) is responsible for the generation of amyloid-β (Aβ) fragments in the brain and is therefore proposed as a therapeutic target for Alzheimer’s disease (AD). Currently BACE1 inhibitors, most of which lack selectivity over BACE2, have demonstrated efficacious reduction of amyloid-β peptides in animals and the CSF of humans. BACE2 knock-out mice have a deficiency in PMEL17 proteolytic processing leading to impaired melanin storage and hair depigmentation. Here, we confirm BACE2-mediated inhibition of PMEL17 proteolytic processing *in vitro* in mouse and human melanocytes. Furthermore, we show that wildtype as well as *bace2*^+/−^ and *bace2*^−/−^ mice treated with a potent dual BACE1/BACE2 inhibitor NB-360 display dose-dependent appearance of irreversibly depigmented hair. Retinal pigmented epithelium showed no morphological changes. Our data demonstrates that BACE2 as well as additional BACE1 inhibition affects melanosome maturation and induces hair depigmentation in mice.

Skin melanocytes are responsible for synthesizing melanins in melanosomes to protect keratinocytes from UV radiation whereas melanin produced by hair follicle melanocytes are involved in hair pigmentation^1^. Hair follicle melanocyte precursor cells migrate from neuronal crests during embryogenesis into the skin area and then proliferate in clones^2^,^3^,^4^. The produced melanin pigments are integrated progressively in the growing hairs, that being only in the anagen phase. Hair cycle phases and lengths are different between mammals and usually occur in waves over the body of young mice^5^. Melanin producing cells are also present in other organs and structures, such as the inner ear, the leptomeninges and the eye (uvea with choroid, ciliary body, iris stroma and the retina with the retina pigmented epithelium (RPE)). Melanocytes populating the RPE have a different origin from skin and hair melanocytes; they are derived from the optic cup of the developing forebrain. Finally, important differences in melanogenesis between hair melanocytes and RPE seem to be present as melanin content and the transcription factor network controlling pigment production differs substantially^6^,^7^.

The diversity in hair pigmentation between individuals and populations results from differences in the ratio of red/yellow pheomelanins and black/brown eumelanins. PMEL (Pigment cell-specific melanocyte protein also known as PMEL17, Silver, SILV, gp100) is a transmembrane glycoprotein solely expressed in melanocytes and is involved in eumelanin synthesis^8^. PMEL17 fibrils act as functional and non-pathological amyloids to sequester highly reactive melanin intermediates^9^,^10^. PMEL17 is a highly conserved protein amongst vertebrates. Several mutations in the mouse, chicken, zebrafish, dog, cow and horse as well as an inactivation of PMEL17 in mice have been linked with hypopigmentation^11^,^12^,^13^,^14^,^15^,^16^,^17^,^18^,^19^. However, in humans there are no reported PMEL17 mutations linked to a disease or pigmentation differences.

It has been shown that the proteolytic processing of PMEL17, reminiscent of amyloid precursor protein (APP) processing, by proprotein convertase, metalloproteinase, γ-secretase and BACE2 and subsequent fibril formations are important for melanosome maturation^20^,^21^ as well as for melanocyte migration in zebrafish^22^. BACE2 is the closest homologue of BACE1 and shares 64% amino acid similarity with BACE1. Both are transmembrane aspartic proteases with high conservation of essential structural features. BACE1, a β-secretase, is involved in concert with γ-secretase in generating the amyloidogenic Aβ from APP^23^ and thereby initiates downstream formation of amyloid-β aggregates and plaques in brain, one of the pathological hallmarks of AD. The “amyloid cascade” hypothesis for AD, the most prevalent age-related dementia affecting currently more than 24 million patients worldwide^24^, states that an imbalance between formation and elimination of Aβ peptide is the initial event in the pathogenesis of AD^25^, marking BACE1 a promising drug target to prevent the generation of Aβ peptides^26^. Whether or not a clinical BACE1 inhibitor need to be selective over BACE2 is currently unknown. While BACE1 is expressed in brain, BACE2 is expressed at lower levels in most peripheral adult tissues^27^ and its role is largely unidentified. BACE2 is reported to be highly expressed in pancreatic endocrine β-cells^28^, and it has been proposed that BACE2-mediated shedding of the transmembrane protein TMEM27 which regulates beta cell mass and controls glucose homeostasis. BACE2 knock-out mice are reported to be viable, fertile and do not display any gross physical or behavioral abnormalities^29^, while certain strains of BACE1 knock-out mice display an enhanced lethality^29^ and retinal pathology^30^. A recent publication on the phenotype of BACE2 knock-out mice^30^, however, showed a highly disorganized choroid of the retina. Furthermore, BACE2 knock-out and double BACE1/BACE2 knock-outs are reported to have slightly lighter hair color, likely due to defect in cleavage of transmembrane protein PMEL17 expressed in skin melanocytes^21^. Rochin *et al.* showed data that the lack of BACE2 triggers PMEL17 misprocessing, leading to melanosome maturation deficits and finally hair hypopigmentation^21^.

In this study, we show that BACE2 but not BACE1 inhibition alters PMEL17 processing and melanin content in human and mouse melanocytes *in vitro*. Furthermore, C57BL/6 treated chronically (2–7 weeks) as well as subchronically (5 days) with a dual equipotent BACE1/BACE2 inhibitor (NB-360^31^) displayed dose- and exposure-dependent, progressive, irreversible patchy hair depigmentation. The pigmentation effects were reversible only after plucking of older hairs enabling the growth of new black pigmented hairs. The reduction in melanin pigments was observed histologically only in anagen (growing) hairs and no changes were observed in skin, telogen (resting) hairs or skin melanocytes or in the eyes. Finally, we show that BACE2 knock-outs have slightly less melanin pigments in hair shafts and uvea of the eye compared to wildtype, and are more sensitive to hair depigmentation with the treatment of the dual equipotent BACE1/BACE2 inhibitor NB-360. These results underscore the importance of BACE2 as well as BACE1 in the hair pigmentation process.

## Results

### BACE2 but not BACE1 inhibition blocks PMEL17 processing and reduces melanin production in mouse and human melanocytes *in vitro*

**Figure 1:**
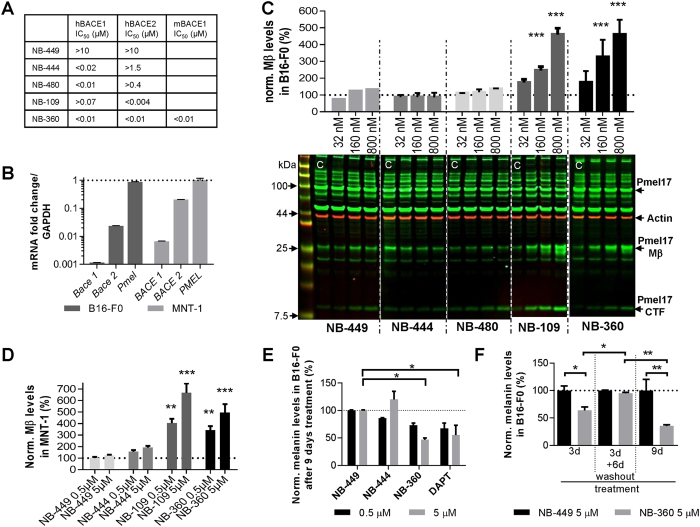
BACE2 but not BACE1 inhibition in B16-F0 and MNT1 cells alters PMEL17 processing and reduces melanin production. (**A**) Pharmacological data on potencies on BACE1 and BACE2 in an enzyme inhibition assay of the tested compounds. (h: human; m: mouse). (**B**) mRNA expression in B16-F0 and MNT-1 cells of BACE1, BACE2 and PMEL; normalized to GAPDH. Data is shown as mean ± SD. (**C**) PMEL17 processing analyses in B16-F0 treated cells with compounds of different selectivity to BACE1 and BACE2. PMEL17 full length as well as the proteolytic cleavage products thereof, PMEL17 Mβ and PMEL17 CTF (C-terminal fragment) are indicated. Bar graphs represent the quantification of Mβ levels detected in immunoblots normalized to DMSO treatment alone in %. The equipotent BACE1/BACE2 inhibitor NB-360 displays a dose-dependent increase of Mβ levels. PMEL17 is proteolytically cleaved first by proprotein convertase to Mα and Mβ fragments. The latter Mβ is processed further by S2 protease to CTF which is a target for γ-secretase. For all conditions n = 2 except for NB-449 (n = 1). Statistics: Holm-Sidak’s multiple comparison test (***p < 0.001). Data is shown as mean ± SEM. (**D**) PMEL17 processing analyses in MNT-1 cells treated with compounds of different selectivity to BACE1 and BACE2. Bar graphs represent the quantification of Mβ levels detected in immunoblots normalized to DMSO treatment alone in %. The equipotent BACE1/BACE2 inhibitor NB-360 displays a dose-dependent increase of Mβ levels. For all conditions n = 3. Statistics: Holm-Sidak’s multiple comparison test (*p < 0.05, ***p < 0.001). Data is shown as mean ± SEM. (**E**) Melanin content in B16-F0 cells after 9 days treatment with the indicated inhibitors at two different concentrations, normalized to NB-449 in %. For all conditions n = 2. Statistics: Holm-Sidak’s multiple comparison test (*p < 0.05). Data is shown as mean ± SEM. (**F**) Melanin content in B16-F0 cells after 3-day treatment, 3-day treatment followed by a subsequent washout for 6 days and 9-day treatment. For all conditions n = 2. Statistics: Newman-Keuls multiple comparison test between the different conditions; Uncorrected Fisher’s LSD within the same condition (*p < 0.05, **p < 0.01). Data is shown as mean ± SEM.

Mouse B16-F0 (mouse melanoma cells) and human MNT-1 (pigmented human melanoma cells) cells were used to determine the extent of PMEL17 processing and melanin production after treatment with different BACE (Fig. 1A) and γ-secretase inhibitors. The expression levels of PMEL17, BACE1 and BACE2 were analyzed in untreated B16-F0 and MNT-1 cells (Fig. 1B). GAPDH mRNA was used as reference. The expression level of PMEL17 was comparable to GAPDH in both cell lines tested. BACE1 and BACE2 are expressed in B16-F0 and MNT-1 suggesting that both cell lines are suited for investigating the effects of BACE inhibitors on PMEL17 processing. However, BACE1 expression was substantially lower than BACE2 in both cell lines.

Cellular viability of treatment with different BACE (Fig. 1A) and γ-secretase inhibitors was estimated by measuring the intracellular ATP levels (Supp. Fig. 1). Both mouse and human melanocytes were treated with increasing concentrations of the compounds (0.2 μM–100 μM) for 24 h. Measurement of ATP levels in mouse melanocytes revealed that the non-cytotoxic threshold concentration was 25 μM for all inhibitors (retained >80% of ATP compared to control). The human melanocytes (MNT-1) displayed a slightly higher non-cytotoxic threshold concentration of 50 μM.

PMEL17 processing in B16-F0 and MNT-1 cells were analyzed after treatment with different BACE inhibitors (Fig. 1C,D), with different potencies against BACE1 and BACE2. PMEL17 is proteolytically processed to give rise to a large Mα and a smaller Mβ fragment by proprotein convertase, whereas the Mβ fragment is further cleaved by BACE2 to the C-terminal fragment (CTF), which is a substrate for γ-secretase^20^. To compare the effect of BACE inhibitors on PMEL17 processing, B16-F0 and MNT-1 cells were treated with different BACE1 and BACE2 selective compounds and with a weakly active diastereomer of NB-360 (NB-449) (Fig. 1A) at three (B16-F0: 0.032 μM; 0.16 μM; 0.8 μM) or two (MNT-1: 0.5 μM and 5 μM) concentrations over 24 h. Specificity of the anti-melanoma PMEL17 antibody recognizing different proteolytic fragments of mouse and human PMEL17 and γ-secretase dependency of PMEL17 processing was confirmed (Supp. Figs 2 and 3). Immunoblot analyses of PMEL17 processing revealed a dose-dependent significant accumulation of Mβ fragment for the potent, dual BACE1/BACE2 inhibitor NB-360^31^ as well as for the more BACE2-selective inhibitor NB-109 whereas the other more BACE1-selective (NB-444, NB-480) or only weakly active (NB-449) compounds displayed no effect on both mouse and human PMEL17 processing (Fig. 1C,D).There was no change in total PMEL17. Parallel to Mβ the CTF fragment was also increased with the dual BACE1/BACE2 inhibitor NB-360 (in MNT-1: Supp. Fig. 3; in B16-F0: Fig. 1C and Supp. Fig. 2) and the more BACE2-selective inhibitor NB-109 (Fig. 1C). Taken together, these results suggest that BACE2 is a key player in mouse and human PMEL17 processing.

Treatment of mouse and human melanocytes with BACE inhibitors at the non-cytotoxic concentrations of 0.5 and 5 μM for 24 h did not have any effects on the melanin content compared to untreated control (data not shown). However upon prolonging the treatment duration for 3–9 days, significant differences in the melanin content were observed (Fig. 1E,F). Reduction of melanin pigment was obvious after treatment with the potent dual BACE1/BACE2 inhibitor NB-360 as well as with the γ-secretase inhibitor DAPT at 5 μM (Fig. 1E). Additionally, the weakly active isomer NB-449 and the more BACE1-selective inhibitor NB-444 had no significant effect on melanin levels (Fig. 1E). Reversibility of BACE inhibition on melanin production in B16-F0 cells could be demonstrated (Fig. 1F). Mouse melanocytes were initially treated for 3 days, followed by a drug-withdrawal phase of 6 days after which the melanin content was measured (Fig. 1F). Melanin content was significantly reduced after 3 days of treatment with NB-360 but returned to control levels after the 6-day recovery period. This can be explained by the fact that the cells are constantly under proliferation *in vitro*. In contrast, prolonged treatment for 9 days with NB-360 drastically reduced the melanin levels to approximately 40% of control. Taken together, these results highlight the involvement of BACE2 in melanogenesis in mouse melanocytes.

### Chronic BACE1/BACE2 inhibitor treatment in mice leads to an exposure-dependent and irreversible hair depigmentation but is devoid of any morphological eye changes

**Figure 2:**
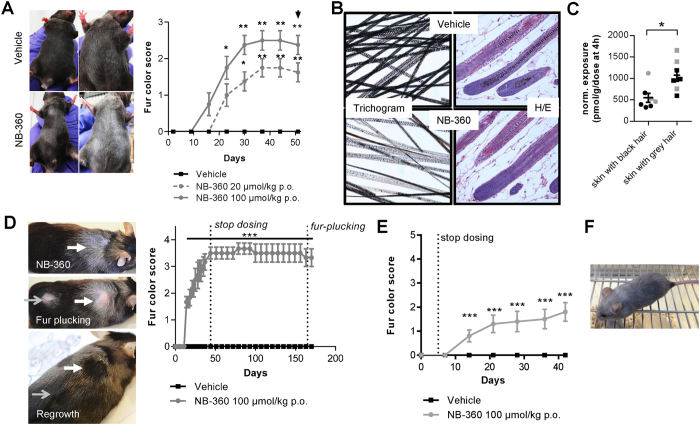
BACE inhibition induces hair depigmentation in mice. (**A**) Example photos of vehicle and NB-360-treated mice. Fur color scores of chronically treated male C57BL/6J mice for 8 weeks with NB-360. Dose-dependent sustained hair depigmentation on the ventral part was obvious after 15 days of treatment for the high dose (100μmol/kg) and 25 days for the low dose (20μmol/kg). In some animals the depigmentation was widespread and extensive while in others it was patchier. In this experiment only the ventral body was affected while the dorsal part showed no change. Statistics: Kruskal-Wallis with Dunn’s post-hoc (*p < 0.05, **p < 0.01 versus vehicle). Vehicle: n = 8, NB-360: each n = 8. Black arrow: final scoring was performed blinded. (**B**) Left: Hair depigmentation assessment by trichoscopy. Hair melanin content was markedly reduced in female wildtype C57BL/6 littermates of the APP51 line treated for 6 weeks with 100 μmol/kg NB-360. Right: Hematoxylin/eosin histopathology of anagen hair follicles showing decreased pigment content in NB-360 treated mice (100μmol/kg) (**C**) Normalized NB-360 black and grey hair skin exposure determination from experiment shown in (A) (normalized to dose at 4 h in pmol/g). Grey symbols: High dose group (100 μmol/kg), Black symbols: low dose group (20μmol/kg). Statistics: two-tailed unpaired t-test (*p < 0.05). (**D**) Fur color score of chronically treated female wildtype C57BL/6 littermates of the APP51 line for 44 days with subsequent washout of 98 days, fur plucking and observation without treatment for further 100 days. Example photos of NB-360 treated mice before treatment stop, after grey (white bold arrows) and black (grey arrows) fur plucking and after hair regrowth. No fur score change was seen during the washout phase, but regrowth of black hair appeared after hair plucking. In this experiment the dorsal part of the mice was more affected. (**E**) Fur color scores of 5-day orally treated male C57BL/6J mice with 100μmol/kg NB-360. After treatment stop the animals were further monitored. Hair depigmentation affecting ventral and dorsal part of the body was again obvious after 2 weeks. (**F**) Animals (APP23 and APPPS1) treated for 6 months with a high dose of NB-360 (food pellet dosing, 0.5 g/kg) were completely grey. For all graphs: Data is shown as mean ± SEM.

Mice were treated chronically with the dual BACE1/BACE2 inhibitor NB-360 (Fig. 1A) to investigate long-term effects on hair depigmentation and eye pathology. Several independent experiments with C57BL/6 were conducted with NB-360 at different doses, selected to achieve a substantial pharmacodynamic effect of NB-360 on Aβ lowering in the brain of male C57BL/6J mice (Supp. Fig. 4A) in the chronic treatment paradigm. Exposure of NB-360 was dose-dependent, and reduced Aβ levels substantially at 4h post dose (20 μmol/kg: 13%, 100 μmol/kg: 2%; relative to vehicle). Even 24 h after end of dosing, a slight reduction in the Aβ levels was observed (20 μmol/kg: 95%, 100 μmol/kg: 53%; normalized to vehicle), in agreement with the lower compound levels in the brain 24 h post dose (Supp. Fig. 4A). At 4 h after the last dose, NB-360 levels in the brain (9405 ± 439 pmol/ml, n = 4) were 4.6-fold higher than blood (2038 ± 246 pmol/ml, n = 4), whereas skin (43798 ± 3580 pmol/ml, n = 4) exhibited substantially higher exposure (21.5-fold) at the highest dose of 100 μmol/kg (Supp. Fig. 4B). Compound levels were dose proportional and decreased substantially 24 h post dose (brain: 139 ± 23 pmol/ml, n = 4; blood: 67 ± 12 pmol/ml, n = 4; skin: 1683 ± 290 pmol/ml, n = 4 for the highest dose of 100 μmol/kg) indicating no accumulation in the skin despite high exposure. Male C57BL/6 mice treated chronically at a dose of 100 μmol/kg NB-360 displayed a slight increase in body weight (approx. 5%) compared to vehicle controls (Supp. Fig. 5). Male C57BL/6J mice (Charles River Laboratories, France) chronically treated (8 weeks) either with 20 μmol/kg or 100 μmol/kg of NB-360 showed a dose-dependent hair depigmentation on the ventral part of the body (Fig. 2A). Vehicle treated control mice did not show any hair color changes. The hair depigmentation at the high dose was more severe (larger grey patches) as indicated by a higher subjective fur color score (scoring of the % of body covered with grey fur, see material and methods) and was also obvious at an earlier time-point (around 2 weeks after start of treatment). In addition, the hair depigmentation reached a maximum and plateaued for both of the doses; there was no further increase of the size of existing grey patches as well as no occurrence of new grey patches between treatment day 37 and end of the study at 51 days (Fig. 2A). The depigmentation was confirmed in a trichogram analysis where hair was plucked from the carcass from pigmented and non-pigmented areas of the body of individual mice and examined microscopically (Fig. 2B). Histopathology analyses of anagen skin hair follicles also revealed an apparent reduction in melanin content (Fig. 2B). Importantly, no other morphological difference of hair follicles could be observed after chronic NB-360 treatment. Interestingly, in this experiment of 8-week NB-360 treated male C57BL/6J mice it was the ventral part of the body that mostly displayed a hair depigmentation whereas the dorsal part of the body was spared. NB-360 levels were measured from skin with black and grey hair and a nearly 2-fold difference in NB-360 levels between black and grey hair areas could be observed (Fig. 2C). Thus, the specific occurrence of grey hair on the ventral part of the body after NB-360 treatment was related to compound level and indicates that the observed depigmentation was dependent on NB-360 exposure in skin.

Subsequently, the reversibility of hair depigmentation was investigated in female C57BL/6Npa (Novartis C57BL/6 strain, littermate wildtypes from the APP51 line) which were treated with 100 μmol/kg of NB-360 for 5 weeks (Fig. 2D). Again, hair depigmentation was obvious after 2 weeks as in the experiment shown in Fig. 2A. However, in this C57BL/6 strain, the hair depigmentation was only visible on the dorsal part of the body, whereas the ventral part was spared. This is in sharp contrast to the experiment shown in Fig. 2A for male C57BL7/6J with only an involvement of the ventral part of the body. As the doses and duration of NB-360 were similar in both experiments only a gender or, a sub-strain difference, affecting skin compound exposures or melanogenesis could account for the different localization of grey hair. Despite a recovery phase of approx. 100 days (>2 hair cycles) no reversal of fur color (grey to black) could be detected (Fig. 2D). Subsequently, the hair was plucked from all the animals at a few sites of grey and normal black hair (Fig. 2D). The new hair that regrew was normally pigmented in both the sites of plucked grey hair as well as black hair, while the unplucked grey hair remained unchanged. This indicates a long retention time of the compound in the hair or melanocytes and that it needs a new proliferation phase of hair bulbs in absence of the compound to establish normal pigmented fur.

Furthermore, due to the irreversibility of hair depigmentation after NB-360 treatment (Fig. 2D), male C57BL/6J (Charles River Laboratories, France) were orally dosed with 100 μmol/kg sub-chronically for only 5 days to see if short compound exposures were sufficient to induce hair depigmentation (Fig. 2E). Interestingly, hair depigmentation was again obvious after 2 weeks, mainly on the ventral part of the body and progressed slowly indicating that even a short high exposure to BACE1/BACE2 inhibitors changes melanocyte function irreversibly.

Long-term treatments with the BACE1/BACE2 dual inhibitor NB-360 in different APP transgenic lines also resulted in hair depigmentation. APPPS1 mice treated for 6 months with NB-360 showed extensive hair depigmentation eventually affecting the whole body (Fig. 2F), which was different to the extent observed in C57BL/6. The first grey spots appeared after 16 days on average in female APPPS1 (1.5-month-old at start of experiment) in the head region whereas for males the belly was affected first after 49 days. Similar results were obtained for APP23 males (15-month-old at start of experiment) treated for 6 months with NB-360, where the first appearance of grey spots was obvious after 23 days on average on the belly or the back. In female APP51 (12.5-month-old at start of experiment) grey spots were visible already after 13 days on average at the same time on the back and the belly. Thus, after prolonged treatment of APP transgenics the whole body, ventral as well as dorsal part, turned completely grey. Some skin areas were affected by BACE inhibition early on, whereas other skin areas display changes later according to the appearance of hair cycle waves. Skin exposure of NB-360 was not measured in APP23 and APPPS1. In summary, the hair depigmentation induced by NB-360, using the same dose across experiments was observed in all mice studied, but the time of onset, the localization, and the extent was gender-, genotype- and strain-dependent.

Importantly, no morphological changes in other pigmented organs could be observed. In particular, the melanin pigments in the uvea and thickness of the retina was unchanged in NB-360 treated animals (female wildtype C57BL/6 mice of the APP51 line) for 6 weeks compared to controls and there was no sign of lysosomal perturbation, lipofuscin accumulation or other changes in the RPE (Supp. Fig. 6).

Moreover, electron microscopic evaluation of the RPE and Bruch’s membrane in 6-week vehicle and NB-360-treated mice (female wildtype C57BL/6 mice of the APP51 line) showed normal ultrastructural appearance, in contrast to data from BACE1 knock-outs or mice treated with the BACE inhibitor AMG-8718^30^,^32^. No phagolysosomal pigment changes or abnormal melanosome morphology were detectable in the RPE (Supp. Fig. 7).

### *bace2* ^−/−^ but not *bace1* ^−/−^ mice exhibit reduced melanin in hair shafts and uvea, but showed no change in RPE

**Figure 3:**
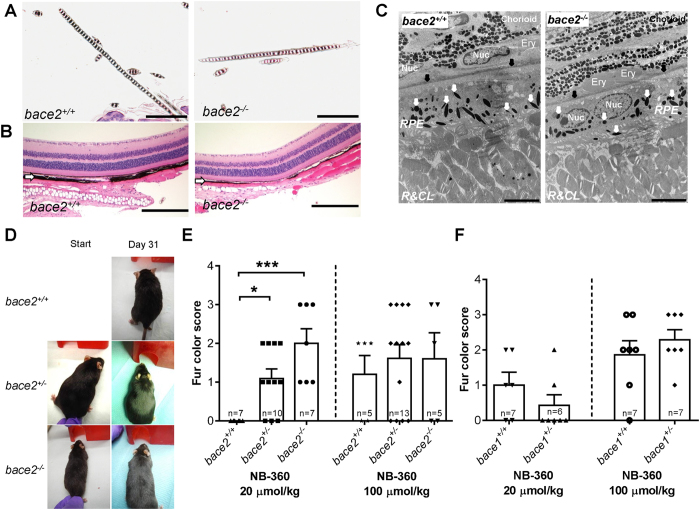
b*ace2*^−/−^ mice show reduced melanin pigment in hair shafts and uvea of the eye and are more sensitive to induction of hair depigmentation by BACE inhibition. (**A**) Hematoxylin and Eosin (HE) staining of hair shafts in 5-6-month old *bace2*^−/−^ compared to littermate wildtye *bace2*^+/+^ male mice. In the skin, hair shafts of all *bace2*^−/−^ mice (8/8) showed reduced melanin content; scale bars −100 μm. (**B**) Hematoxylin and Eosin (HE) staining of the uvea of the eye in 5-6-month old *bace2*^−/−^ compared to littermate wildtye *bace2*^+/+^ male mice. In the uvea, thinning of the choroid and reduced melanin in iris and ciliary body was obvious in 7/8 males and 3/6 females. White arrow: choroid; scale bars −200 μm. (**C**) Ultrastructural analysis of the RPE and choroid in 5-6-month old *bace2*^−/−^ and littermate wildtye *bace2*^+/+^ male mice. White arrow: electron dense pigment granules; black arrows: Bruch’s membrane; Nuc: Nucleus; Ery: erythrocytes, RC&L: rod and cones layer; scale bars −20 μm. (**D**) Example photos of wildtype littermates (*bace2*^+/+^) and BACE2 knock-outs (heterozygous^+/−^, *bace2*^+/−^ and homozygous^*−*/*−*^, *bace2*^−/−^) treated daily with high-dose (100 μmol/kg, p.o.) of NB-360 showing the appearance of grey hair patches, mainly on the dorsal part of the body. (**E**) Fur color score scores of *bace2*^+/+^, *bace2*^+/−^ and *bace2*^−/−^ mice chronically treated for 31 days with NB-360 at 20 μmol/kg or 100 μmol/kg, showing a gene-dose-dependent hair depigmentation effect for the low-dose. Individual results and mean ± SEM are shown. Statistics: Kruskal-Wallis with Dunn’s post-hoc (*p < 0.05, ***p < 0.001 versus *bace2*^+/+^). (**F**) Fur color score scores of chronically treated wildtype littermates (*bace2*^+/+^) and BACE1 heterozygous knock-outs (heterozygous^+/−^, *bace1*^+/−^) for 31 days with NB-360 at 20 μmol/kg and 100 μmol/kg showed an undistinguishable effect on grey hair compared to littermate controls. Individual results and mean ± SEM are shown.

Our BACE1 and BACE2 knock-out mouse (*bace1*^−/−^ and *bace2*^−/−^, respectively) colonies were obtained from the lab of B. De Strooper^29^. We used two different colonies: one colony in a mixed unknown background and one backcrossed to C57BL/6 for at least 6 generations. *bace2*^−/−^ mice did not show any visible differences in color of the fur compared to littermates, even at older ages (>1 year). However, microscopic evaluation revealed changes with minimally reduced melanin pigment present in hair shafts of *bace2*^−/−^ (Fig. 3A), but not *bace2*^+/−^ mice (data not shown) compared to the wildtype controls, similar to other publications using mice from the same source^21^. In 9 out of 16 animals the change was observed in either ventral or dorsal skin but not both sites highlighting the minimal nature of the change. In the eye, changes were observed only in the pigmented uvea structures that consisted of focal areas of thinning of the choroid melanin and reduced melanin in the iris and ciliary body in 7 out of 8 males and 3 out of 6 females analyzed (Fig. 3B). No change could be observed in the RPE by light or fluorescence microscopy (data not shown) as well as in electron microscopic evaluation of the RPE and Bruch’s membrane in *bace2*^−/−^ mice (Fig. 3C). We also investigated our colony of BACE1 knock-outs. Our BACE1 knock-outs were viable, fertile and did not show an increased mortality as previously described for this strain^29^. The only histopathology differences were in the peripheral nerves (data not shown) as previously reported by others in another BACE1 knock-out strain^33^,^34^. Moreover, electron microscopic evaluation of the RPE and Bruch’s membrane in *bace1*^−/−^ mice showed normal ultrastructural appearance; contradicting published observations with another strain of BACE1 knockouts^30^. No phagolysosomal or pigmental changes or abnormal melanosome morphology were detectable in the RPE (Supp. Fig. 8).

### BACE2 gene-dose dependent sensitivity of BACE inhibition-related hair depigmentation. 

To investigate if there was a changed sensitivity to BACE inhibition on hair depigmentation we chronically (31 days) treated aged male and female BACE1 and BACE2 knock-outs (heterozygous^+/−^, *bace2*^+/−^ and *bace1*^+/−^; and homozygous^−/−^, *bace2*^−/−^) with NB-360 (Fig. 3D–F). *bace1*^−/−^ were not tested. It is important to mention, that the *bace2*^+/−^, *bace2*^−/−^ and *bace1*^+/−^ animals used in this experiment were not in a pure C57BL/6 background (in contrast to animals used above for microscopic evaluations), but were of a mixed unknown background. Nevertheless, the high dose of 100 μmol/kg NB-360 again induced a grey hair color (Fig. 3D–F) in all genotypes, albeit more variably than in the previous experiments with the pure C57BL/6 background, as shown in Fig. 2A,D. This could very well be due to the difference in age (>1 year) or the unclear genetic background. Wildtype littermates of *bace2*^−/−^ did not display any hair color change with the low dose of 20μmol/kg, as for C57BL/6 in other experiments, which might be again due to the age or genetic background. However, there was a gene-dose dependent effect of BACE2 on the hair depigmentation observed (Fig. 3E). While *bace2*^+/−^ showed a mild but significant hair depigmentation upon NB-360 treatment, *bace2*^−/−^ displayed a stronger hair color change (Fig. 3D,E). NB-360 did not induce a gene-dose dependent hair depigmentation in *bace1*^+/−^ (Fig. 3F, *bace1*^−/−^ were not tested). Thus, *bace2*^−/−^ and *bace2*^+/−^ were more sensitive to BACE inhibition regarding hair depigmentation than wildtype and *bace1*^+/−^.

In summary, mice treated with a dual BACE1/BACE2 inhibitor developed dose-dependent and irreversible hair depigmentation sparing retina, RPE and other pigmented tissues. *bace2*^−/−^ do not show any visible grey hair macroscopically but upon dual BACE1/BACE2 inhibition readily display hair depigmentation indicating that BACE1 could be involved in melanogenesis.

## Discussion

This study shows for the first time that an equipotent BACE1/BACE2 inhibitor induced dose-dependent hair depigmentation in wildtype as well as in *bace2*^−/−^ mice. The hair depigmentation became visible approximately 2 weeks after the start of dosing, and was observed both after a short 5-day exposure to the BACE1/BACE2 inhibitor NB-360^31^ and after longer periods of daily dosing. Hair pigmentation is tightly linked to the hair cycle. The hair cycle in mice occurs in waves which spreads anterior-posteriorly and ventral-dorsally, so that all follicles in an area are in the same phase of growth; the cycles are influenced by age as well^5^. The appearance of grey hair after approximately 2 weeks fits quite well with the duration of the anagen phase, which is about 17–20 days. In this phase melanocytes proliferate and differentiate; the bulb and lower part of new hair contain high amount of pigment^5^. Hair depigmentation appeared first in patches and was either on the ventral or dorsal surface of the body and in defined skin areas. The hair depigmentation in our mice seemed to be dependent on gender, age, genetic background, dose and treatment duration. In male C57BL/6 the grey patches were visible on the ventral surface while in female C57BL/6 the dorsal surface was affected. However, in other experiments both, the ventral and dorsal part of the body were affected. In addition, aged mice with a mixed genetic background seemed not to be as sensitive as pure C57BL/6 as the low dose of 20 μmol/kg NB-360 did not induce hair depigmentation after 31 days of treatment. Nevertheless, in APP transgenic mice longer treatment durations (>5 months) had a stronger effect on hair depigmentation; the proportion of the body with grey hair increased with prolonged treatment time, resulting ultimately in the appearance of a complete grey-colored mouse which is again in accordance with the occurrence of hair cycles in waves.

Compound exposure in skin seems to be critical for inducing altered melanogenesis in mice. NB-360 exhibited very high skin levels (at least >20-fold over blood). However, there was an approximately 2-fold difference observed between the levels in skin where black and grey hair occurred which seemed to determine hair depigmentation. It could very well be that a high NB-360 exposure early in the treatment paradigm induces hair depigmentation whereas a lower NB-360 dose only does so after extended treatment before reaching the critical threshold exposure. Hence, after 6 months of treatment all of the skin has reached the threshold of NB-360 exposure that was necessary to induce hair depigmentation. The ability of NB-360 to induce hair depigmentation may be related to the free, rather than the total, compound exposure in the target tissue. Very high non-specific binding of NB-360 to skin is expected based on data in other tissues, but it was not possible to determine the free concentration in skin experimentally. Furthermore, two effects may contribute to increase NB-360 concentrations in melanosomes: slightly basic compounds like NB-360 accumulate in acidic organelles such as endosomes and melanosomes, and binding to melanin may further increase the NB-360 concentration within the melanosome. Furthermore, compound accumulation in the skin has not been observed.

An interesting observation is the irreversibility of hair depigmentation. Despite a long washout phase of more than 100 days, which is three times the hair cycle in mice, the grey hair appeared unchanged and normal regrowth of black hair occurred only after hair-plucking, which presumably induced regeneration of melanocytes from melanoblasts. Furthermore, a brief 5-day exposure to NB-360 was enough to induce an irreversible hair depigmentation indicating that BACE1/BACE2 inhibition in hair follicles induces long-lasting changes. This is in stark contrast to *in vitro* findings where a reversibility of melanin production after removing the BACE inhibitor in mouse B16-F0 melanocytes could be observed. However, the highly proliferating B16-F0 cells *in vitro* might not represent fully the complex situation *in vivo*. Although NB-360 skin levels decreased substantially 24 h after the last dose the remaining compound levels in the skin/hair follicle could have been high enough to effectively inhibit BACE. NB-360 levels beyond 24 h have not been analyzed. In addition, BACE inhibition could possibly influence the hair cycle, extend the anagen phase of the hair follicle or even induce epigenetic changes. Further studies are needed to resolve this question.

In *in vitro* studies in mouse and human melanocytes using BACE specific inhibitors, we could show that BACE2 rather than BACE1 inhibition was involved in PMEL17 processing and melanin production. The equipotent BACE1/BACE2 inhibitor NB-360 reduced PMEL17 processing in both mouse and human melanocytes whereas more BACE1 selective inhibitors did not alter PMEL17 cleavage. Thus, our study suggests that mainly BACE2 is involved in the melanogenesis of the hair follicle and that BACE2 inhibition leads to hair depigmentation in mice. Nevertheless, BACE1 is expressed at very low levels in melanocytes whereas BACE2 is expressed much higher at the RNA level. Thus, normally BACE2 seemed to be mainly responsible for PMEL17 processing and melanin production. Our findings confirm previous results showing that BACE2 is involved in PMEL processing and melanocyte function^21^,^22^. Similar to our results, Rochin *et al.*^21^ demonstrated that MNT-1 cells treated with a β-secretase inhibitor showed an increase in the Mβ fragment as well as the CTF fragment. However, this was in contrast to their BACE2 RNAi experiments which induced rather a reduction of the CTF fragment. These results imply a certain involvement of BACE1 in proteolytic activities downstream of Mβ. Important to note is that NB-360 has no activity on inhibiting γ-secretase (data not shown).

The hair depigmentation phenotype has also been observed in mice with an inactivation mutation of *Pmel* (PMEL17^14^). In these mice hair pigmentation is affected, eumelanin is reduced and melanocyte morphology is changed and the phenotype is similar to that observed in the *silver* mouse^35^. However, these effects were rather subtle compared to the PMEL mutations that cause hypopigmentations in the cow, horse and chicken^11^,^12^,^15^,^18^ indicating that the latter mutations rather represent dominant negative forms of PMEL17 with a more dramatic effect on follicular melanocytes. In mice treated with the dual BACE1/BACE2 inhibitor NB-360 an obvious hair depigmentation was detected. Trichoscopic analyses of the grey hairs revealed substantial decrease in hair pigmentation. This visible hair depigmentation is in contrast to the PMEL knock-out data, where only subtle visible hair depigmentation could be detected^14^. A possible explanation could be that the complete and constitutive inactivation mutation of PMEL during development triggers compensatory mechanisms. In line with the PMEL knock-outs^14^, our BACE2 knock-out animals, which were not in a pure C57BL/6 background, did not show any visible depigmentation, only upon microscopic inspection was a minimally reduced melanin content in hair shafts obvious, similar to observations described elsewhere^29^. Importantly, heterozygous *bace2*^+/−^ mice did not show any difference in hair pigmentation. However, BACE2 knock-out animals were more sensitive to a low dose of NB-360, showing dose-dependent and a more ready hair depigmentation than the littermate wildtype controls. This indicates that BACE1, although only present at low levels, is able to compensate for the function of BACE2 in melanogenesis if the latter is not present or is reduced. In accordance, heterozygous BACE1 knock-outs were not altered in their sensitivity to BACE inhibition. Furthermore, BACE1 levels in the skin could be altered in the BACE2 knock-out, which we did not analyze. Importantly, BACE2 RNA is normal in BACE1 knock-outs^36^.

Chronic pharmacological dual BACE1/BACE2 inhibition in mice did not induce changes in other pigmented structures, especially the eyes, as has been described elsewhere^30^,^37^. Cai and coworkers described retinal pathology in BACE1 knock-out^30^. In our mice treated for 6 weeks with a high-dose of BACE1/BACE2 inhibitor as well as in BACE1 knock-outs, a detailed analysis of the eye was performed and revealed no change in melanin content, no lysosomal perturbation, lipofuscin accumulation or retinal thinning. However, changes in the eye of BACE2 knock-outs were observed in the uvea only specifically, a thinning of the choroid melanin and reduced melanin in iris and ciliary body, but with no change in the RPE and Bruch’s membrane. These observations in the eye of BACE2 knock-outs differ slightly to other publications^21^,^30^, that describe mainly abnormal melanosome morphology and increased lipofuscin in the RPE as well as a disrupted choroidal pigmented layer. These differences in phenotypes could be a result of age or of the background strain of the animals. Nevertheless, despite these differences in the extent and the exact localization of the pathology, there is consensus that *bace2*^−/−^ mice display alterations in the eye and skin.

PMEL17 has been shown to be required for normal development of the melanosomes in skin, choroid and RPE cells; PMEL knock-out mice showed altered melanosome shape and melanin deposition in RPE^14^. However, this structural change of RPE melanosomes did not impact retinal functionality^14^.

In summary, our study confirms that BACE2 is a dominant player for PMEL17 processing and melanin synthesis in hair melanocytes. Most probably due to its low expression, BACE1 plays a minor role under normal conditions. However, under conditions of substantially reduced or absent BACE2 activity, BACE1 may be able to compensate for BACE2. Co-inhibition of BACE1 and BACE2 therefore strongly affects hair depigmentation.

While this pharmacologically induced “grey hair” phenotype is easily detected, it is still unknown whether or not it is an indicator of alterations in melanin processing in other pigmented organs. No such changes were observed with NB-360, inhibiting BACE1 and BACE2 with the same potency, nor in BACE1 knock-out mice. Since melanin exerts important functions including filtering UV light, scavenging reactive oxygen species and iron storage, perturbations in melanin production or storage could potentially have detrimental effects. Long term treatment with BACE inhibitors is anticipated in Alzheimer’s disease^26^,^38^ and BACE inhibitors should therefore be carefully selected based on their selectivity and tissue distribution profile.

## Materials and Methods

### Ethics Statement

All experiments were carried out in accordance with authorization guidelines of the Swiss Federal and Cantonal veterinary offices for care and use of laboratory animals and the veterinary office regulations of Baden-Württemberg (Germany) and approved by the local Animal Care and Use Committees. Studies described in this report were approved by the Swiss Cantonal Veterinary Authority of Basel City, Switzerland, under the license number 1094.

### Animals

C57BL/6J animals were source from Charles River Laboratories, France. C57BL/6 control littermates from the APP51 line were bred at Novartis Pharma AG, Basel, Switzerland.

APP23, APPS1, and APP51 mouse lines used all express human APP751 under the control of the murine Thy1-promoter, resulting in neuron-specific expression. APP23 mice^39^ express APP with the K670M/N671L “Swedish” mutation. The mice have been backcrossed with C57BL/6J mice for 20 generations (C57BL/6J- Tg(Thy1-APP_K670N;M671L_)23). APP23 mice are currently bred at the Hertie Institute for Clinical Brain Research (Tübingen, Germany) and overexpress human APP approximately 7 times over endogenous APP and first develop individual Aβ-amyloid plaques in the neocortex at 7 months (females) or 9 months of age (males). For this study male APP23 mice between 15 and 21 months of age were used.

Male and female APPPS1 mice of ages 1.5 to 7.5 months were bred at the Hertie Institute for Clinical Brain Research (Tübingen, Germany). APPPS1 mice have been initially generated and maintained on a C57BL/6 background and co-express K670M/N671L-mutated APP and L166P-mutated presenilin 1 (PS1) (C57BL/6J-TgN(APPswe and PS1 166)21)^40^. The mice first develop Aβ plaques after 6 weeks of age, and no effect of gender was found.

Female 12.5 to 13.5 month-old APP51/16 mice (C57BL/6J-TgN(Thy1-APP)51; APP51) were bred on a C57BL/6J background at the Hertie Institute for Clinical Brain Research (Tübingen, Germany). APP51 mice^41^ express human wild type APP at about 7–10-fold over endogenous APP and develop the first plaques between 12 and 15 months of age. All mice were kept under specific pathogen-free conditions.

BACE1 (B6,129T2-TgH(Bace1)1Goe) and BACE2 (B6,129P2-TgH(Bace2)1Leu) knock-out mice were received from the lab of B. De Strooper^29^, with unknown genetic background. Animals were black. For the pharmacological experiment with NB-360 this colony was used. For the detailed histo-pathological caharcaterization knock-outs were backcrossed at least 6 generation into C57BL/6 (>98%).

Upon arrival in the laboratory, single-housed mice were maintained under standard conditions in temperature and humidity control rooms under a 12/12 light/dark schedule, with lights on at 06:00 hr. Cage bedding was sawdust, and a red Pespex mouse-house (Nalgene^®^), nesting materials (Nestlet^®^) and a wooden gnawing-block were supplied in each cage. Either standard laboratory rodent food (C57BL/6) or food pellets containing NB-360 (APPPS1, APP23, APP51) and tap water were available *ad libitum*.

### Compound form, formulation and dosing

Crystalline NB-360 was formulated as a suspension. Vehicle or compound were given *per os* in a volume of 10 ml/kg once daily (mornings). Vehicle: 0.1% Tween80 in 0.5% Methylcellulose in water. All suspensions were homogeneous upon visual inspection. Particle size was in the low micrometer range for all suspensions.

NB-360 was dosed in food pellets (0.5 g/kg) to treat APPPS1, APP23, and APP51 mice. Food pellets (KLIBA NAFAG) were produced at Provimi Kliba SA, Kaiseraugst, Switzerland.

### Fur color scoring

Subjective scoring of any hair color changes was performed once weekly. Scores (% of body with grey fur): 0: No change; 1: Spots; 2: >30%; 3: >50%; 4: >75%; 5: 100%. Animals were photo-documented when a fur color change was observed.

### Trichogram

Mouse hair was placed on glass slides with some drops of oil, cover slipped and examined microscopically.

### Histology on eye samples

Both eyes were collected and either fixed in Davidson’s fixative or OCT embedded. From two mice/group retina only was collected instead of the entire left eye. Subsequently the Davidson’s fixed eye samples were paraffin embedded and further processed while the OCT embedded samples were stored at −80 °C.

Immunohistochemistry and immunofluorescence were performed using antibodies directed against LAMP2 (1:200, rabbit polyclonal IgG, ThermoFisher Scientific, 51-2200) and Rhodopsin (1:1000, rabbit polyclonal IgG, abcam, ab104760). Lipofuscin accumulation was assessed by comparing Rhodopsin fluorescence to autofluorescence. LAMP-2 immunohistochemistry was performed using the fully automated instrument Discovery XT^®^ (Ventana Medical Systems Inc., Switzerland). All chemicals were provided by Ventana Medical Systems Inc.

Briefly, sample slides from Davidson-fixed paraffin embedded tissue were deparaffinized and rehydrated under solvent-free conditions using EZprep™ solution for 8 minutes at 75 °C. Depigmentation was performed using a solution of H_2_O_2_ 3% (Merck, Germany) at 55 °C during 1 hour. Subsequently, heat induced epitope retrieval pretreatment was performed by successive cycles (4×) at 100 °C for 4 minutes in a Tris-EDTA based buffer optimized for the Discovery XT^®^ instrument (CC1 solution). Before applying the primary antibody, the slides were blocked using 1x Casein solution in PBS (BioFX laboratories, Catalog number PBSC-0100-5×) for 32 minutes at room temperature to avoid background. Endogenous avidin/biotin activity was also quenched by using Ventana A/B blocking reagents for 4 minutes each. A short post-fixation using glutaraldehyde at 0.05% was done before applying a biotin conjugated secondary antibody (Jackson Laboratories, Switzerland) diluted at 1/500 in Discovery antibody diluent and incubated for 16 min. Detection of the biotin was performed with the RedMap streptavidin-alkaline phosphatase detection system (Ventana Medical Systems Inc., Switzerland). The detection system was used according to the manufacturer’s recommendations. Counterstaining with Hematoxylin II and Bluing reagent was performed for 2 × 8 min. Sections were dehydrated and covered using Pertex.

For the fluorescence detection of Rhodopsin the above described protocol was applied except for antibody dilution (1:1000, goat anti-rabbit Alexa fluor 647 conjugated).

Detection was performed manually using goat anti-rabbit Alexa fluor conjugated antibodies (Life Technologies, Switzerland), diluted 1/200 in Amplifying antibody dilution buffer (Cat No. AA4, ProHisto, USA) and incubated 30 minutes at room temperature.

The nuclei were detected using ProLong^®^ Gold Antifade Reagent with DAPI (Cat. No. P36931, Molecular Probes/Life Technologies, Switzerland) and covered with ProLong^®^ Gold Antifade Reagent (Cat. No. P36934 Molecular Probes/Life Technologies, Switzerland).

Lipofuscin accumulation was assessed by comparing Rhodopsin fluorescence to autofluorescence. The specific fluorescence signal was detected using the Leica SP5 confocal microscope (Leica, Germany). Assessment of lipofuscin deposition and distribution in the RPE was performed by recording its autofluorescence at 488 nm excitation wave length using Leica SP5 confocal microscope (Leica AG, Germany).

### Electron microscopy on eye samples

The right eyes from 1 vehicle-treated mouse and two compound-treated mice were sampled and immediately fixed in 3% glutaraldehyde and 0.1 M phosphate buffer, pH 7.4, for 24h at 4 °C. A post-fixation was performed in 1% OsO4 in 0.1 M phosphate buffer, pH 7.4, for 1 h at 4 °C. The eyes were subsequently trimmed and dehydrated in graded acetone solutions. A standard Epon embedding procedure was applied, ultrathin sections were cut and assessed using the Tecnai Spirit Transmission Electron Microscope (FEI, Switzerland). For each investigated retina the RPE, Bruch’s membrane were evaluated. For each retina at least 3 different locations were examined.

### *Ex-vivo* samples and sample harvest methodology

Blood samples were used to analyze whole-blood compound levels and were obtained either from tail vein during the in-life part into EDTA tubes (CB300, Sarstedt, Germany) or from trunk blood on the day of necropsy into EDTA Eppendorf tubes (Milian SA, CatNoTOM-14, Fisher Scientific, Wohlen, Switzerland), frozen on dry ice and stored at −80 °C until analysis.

The brain was removed immediately after decapitation, rinsed with saline and sectioned sagitally down the midline. The left half of the cerebellum was used to analyze compound level and was placed into a glass tube (Chromacol, 125 × 5-SV T051, Welwyn Garden City, United Kingdom), weighed and frozen in dry-ice. The left half of the forebrain (without olfactory bulb) was used for Aβ analysis, and was frozen on a metal plate on dry ice and placed into protein Lo-bind tube (003 0108.116, Eppendorf, Hamburg, Germany).

Ventral and dorsal skin were taken to analyze compound level, weighed and frozen on dry-ice.

### Analysis of compound levels

NB-360 levels in biological samples were quantified in blood, brain and skin by liquid chromatography/tandem mass spectrometry (HPLC/MS/MS). Brain samples were mixed with 2 volumes of KH_2_PO_4_ buffer and homogenized using the Covaris^®^ device. Skin samples were mixed with approx. 6-fold volumes of methanol/water and homogenized using Precellys tubes. Either 30 μL of blood, brain or skin homogenate were spiked with a structurally related internal standard and subsequently mixed with an at least 6-fold excess volume acetonitrile for protein precipitation. The supernatant was injected directly into the LC/MS/MS system for analysis.

### Brain homogenization

Frozen mouse forebrains were weighed and homogenized in 9 volumes (w/v) of ice-cold TBS-Complete (20 mM Tris-HCl pH 7.4, 137 mM NaCl, 1× Complete [Protease Inhibitor Cocktail Tablets: 11836145, Roche Diagnostics GmbH, Penzberg, Germany]) by sonication (90% duty cycle, output control 5, 40–55 pulses, [Sonifier 450, Branson]). After homogenization several 50 μl aliquots were prepared for analysis and were stored at −80 °C.

### Determination of Aβ40 in mouse brain

Human Aβ peptide (1–40) trifluoroacetate salt (H 1194.1000, Bachem, Bubendorf, Switzerland) was used as standard. Endogenous Abeta40 in mice was determined with the Meso Scale Discovery (MSD) 96-well MULTI-ARRAY human/rodent (4G8) Aβ40 Ultrasensitive Assay (#K110FTE-3, Meso Scale Discovery, Gaithersburg, USA). The assay was done according to the manufacturer’s instructions except for the calibration curve and the sample preparations. TritonX-100 (TX-100) soluble Aβ40 was extracted from forebrain with 1% TX-100 using a 50 μl aliquot of each 1:10 forebrain homogenate, mixed with 50 μl 2% TX-100 in TBS complete (20 mM Tris-HCl pH 7.4, 137 mM NaCl, 1× Complete [Protease Inhibitor Cocktail Tablets: 11836145, Roche Diagnostics GmbH, Penzberg Germany]) to reach a final concentration of 1% TX-100 and a 1:20 forebrain dilution. The samples were incubated for 15 min on ice and vortexed every 5 min. The samples were ultra-centrifuged (100000×g, 4 °C, 15 min) and 50 μl of the clear supernatants were transferred to fresh tubes. For the Aβ40 assay the supernatants were further diluted 1:5 in 3% Blocker A solution (from kit) to a final forebrain dilution of 1:100 and applied to the plate.

The calibration curve was prepared in a corresponding dilution of 1% Blocker A solution spiked with synthetic Aβ1-40 peptide (1.56–100 pg/ml) except for non-transgenic mouse brain samples: In this case, the calibration curve was prepared in a correspondingly diluted APP knock-out mouse forebrain spiked with synthetic Aβ1–40 peptide (1.56–100 pg/ml). For all samples and standards 25 μl were applied per well. For each determination duplicate wells were done. The mean values from the duplicate wells were used for calculations. Since MSD did not provide quantification software, the relative units for samples and standards were imported into SOFTmax PRO 4.0 for calculation of standard curves and quantification of samples.

### Compounds for *in vitro* usage

NB-360, NB-449, NB-444, NB-480 and NB-109 are products of Novartis Pharma AG. The γ-secretase inhibitor N-[N-(3,5-difluorophenacetyl-L-alanyl)]-S-phenylglycine t-butyl ester (DAPT) was purchased from Sigma (D5942). All compounds were dissolved in DMSO. Stocks of 50 mM solution were prepared and stored as aliquots in −20 °C to reduce several freeze thaw cycles.

### Cell culture systems

Human MNT-1 cells [Cuomo M, 1991] were purchased from MediaPharma, Chieti, Italy. Mouse melanoma B16-F0 cells were bought from ATCC (CRL-6322). The human MNT-1 cells were cultured in Dulbecco’s Modified Eagle’s medium (DMEM, Invitrogen) supplemented with 16% fetal bovine serum (Hyclone), 10% AIM-V medium (Invitrogen), 0.1 mM non-essential amino acid mix (Invitrogen) and 1 mM sodium pyruvate (Invitrogen), and 100 U/ml of penicillin/streptomycin (Invitrogen). B16F0 cells were cultured in DMEM supplemented with 10% Fetal Bovine Serum (FBS) and 100 U/ml of penicillin/streptomycin (Invitrogen). Both cell lines were cultivated at 37 °C, 5% CO_2_ and 98% relative humidity in a tissue culture incubator (model Cytoperm 2, Heraeus Instrument, Hanau, Germany). Cells were passaged once to twice weekly by using Trypsin-EDTA solution.

### Intracellular ATP measurement

Intracellular ATP levels were measured as an indicator of energy homeostasis and cellular viability. Melanocytes were seeded in 96 well plates (B16-F0: 1000 cells/well; MNT-1: 10,000 cells/well). Twenty four hours post-seeding, both cell lines were incubated with increasing concentration of inhibitors over another twenty four hours. ATP measurements were performed using CellTiter-Glo Luminiscent Cell Viability kit (Promega) as described by the manufacturer. Briefly, 100 μl of the CellTiter-Glo Reagent were added to the 100 μl of the medium, placed in a shaker for 2 minutes and incubated at room temperature for 10 minutes to induce complete lysis. The resulting luminescence was measured using the Envision 2104 luminescence plate reader (Perkin Elmer). Data were expressed relative to untreated controls.

### RNA extraction, cDNA synthesis and real-time PCR

Total RNA samples were extracted from cells by using RNeasy Plus Micro Kit (Qiagen #74034). RNA integrity was controlled by running a Bioanalizer profile (Agilent) and by measuring their spectral profile at λ = 230, 260 and 280 nm.

300 ng of total RNA was reverse transcribed into cDNA by using the High Capacity cDNA Archive Kit and according to the manufacturer instructions (Applied Biosystems, cat. No. 4368813).

Real-time PCR was performed by using the TaqMan^®^ fast PCR Master Mix (Applied Biosystems, cat no. 4352042) and the ABI prism™ 7900HT (Applied Biosystems) according to the manufacturer instructions.

Relative quantification of gene expression changes was performed by using the standard curve method and normalized to control samples to generate fold changes values. The housekeeping gene used for comparison and normalization of gene expression data was the GAPDH RNA.

Results were presented in fold change as compared to BACE1 expression.

Taqman assays used to assess gene expression: *PMEL* Mm00498996_m1, *BACE1* Mm00478664_m1, *BACE2* Mm00517138_m1, *PMEL* Hs00173854_m1, *BACE1* Hs01121195_m1, *BACE2* Hs00273238_m1, *18S* Hs99999901_s1 Endogenous control, *GAPDH* Hs02758991_g1 Endogenous control, *GAPDH* Mm99999915_g1 Endogenous control

### Melanin measurement

B16-F0 cells were seeded in 6 well plates (500 cells/well) and left to attach overnight. The following day, melanocytes were treated with different concentrations of the compounds for a total duration of 9 days. After every 3 days, both the compounds and the media were replenished. Melanin concentrations were determined by spectrophotometric measurement of the absorbance at 405 nm (Chu HL 2009; Ishida K 2009; Haywood RM 2006). Briefly, upon completion of the treatment, cells were trypsinized and the melanin content was extracted by incubating the melanocytes with 1N NaOH for 2 hours at 80 °C accompanied by mild shaking. The supernatant was then centrifuged and the absorbance was measured at 405 nm in spectrophotometer (SpectraMax 190, Molecular Devices). Melanin concentrations were then extrapolated using a standard curve (0 – 12.5 μg/ml) obtained with synthetic melanin using SoftMax Pro software. For the standard curve, synthetic melanin (Sigma, Cat. No. M0418, Lot no. 011M1326V) was prepared at 20 mg/ml in 1N NH4OH buffer.

### Cellular treatments

Melanocytes were seeded in 6-well plates. Murine B16-F0 cells were plated at 8 × 10^4^ cells/well and human MNT1 cells were plated at 8 × 10^4^ cells/well for protein extraction and western blot analysis. Twenty four hours post seeding, both cell lines were incubated with increasing concentration of inhibitors over twenty four hours. The cells were washed once with PBS (Invitrogen) and then trypsinized with 222 μl of trypsin (Invitrogen). After trypsinization, the plates were placed on ice and 1778 μl of cold medium was used to stop the action of trypsin. The cell suspensions were transferred in pre-cooled Eppendorf tubes and centrifuged at 4 °C for 7 min at 450g. The pellets were washed with 200 μl of cold PBS to remove debris and remaining media. The pellets were centrifuged once more as described previously. The supernatants were removed and the pellets were lysed on ice as described below.

### Protein extraction, concentration measurement and immunoblotting

After washing the cell pellets, 60 μl of ice cold RIPA buffer (Pierce 89900) including protease and phosphatase inhibitors (Sigma P8340, P2850, P5726-1/100 each) were added to the pellets and the resulting lysates were vortexed shortly. Pellet suspensions were incubated under agitation over 20 min at 4 °C by using a thermomixer (Eppendorf). Pellet suspensions were finally centrifuged (15 min, 16 000×g, 4 °C) and supernatants were collected and stored at –80 °C. An aliquot was subsequently taken to estimate the protein concentration using the MicroBCA Assay Kit (Pierce, #23235) and separately stored at −20 °C. Absorbance was measured at 562 nm (Envision 2104, Perkin Elmer).

SDS-PAGE samples were prepared in NuPAGE 4× buffer (Invitrogen NP0007) and 10× reducing agent (Invitrogen NP0004) or in 6× loading buffer (60 mM Tris pH 6.8, 2% SDS, 9.8% glycerol, bromophenol blue, 0.6 mM DTT). Samples were heated (10 min, 70 °C), centrifuged (3 min, 14 000×g) and resolved on Invitrogen NuPAGE Bis-Tris gels (4–12% or 8% gel, MES running buffer, 160 V, 1 hour). Gels were transferred to nitrocellulose membranes (20 V, 7 min) using the I-Blot system (Invitrogen, #IB1001EU). Membranes were blocked for 1 hour in Odyssey Blocking Buffer (LI-COR 927-40000)/PBS (1:1) and subsequently incubated overnight at 4 °C with primary antibodies (anti-PMEL17, abcam Ab52058, goat polyclonal IgG, 1:250; anti-actin, Sigma A5060, rabbit polyclonal IgG, 1:15000) diluted in Odyssey Blocking Buffer/PBS-Tween 0.1% (1:1). After washing 3 times with PBS-Tween 0.1%, the membranes were incubated for 1 h with secondary antibodies labelled with IRdyes diluted in Odyssey Blocking Buffer/PBS-Tween 0.1% (1:1). The membranes were washed (3 times with PBS-Tween 0.1%, 3 times with PBS) and then dried on absorbent paper before being scanned using the Odyssey Infrared Imaging System (LI-COR).

Integrated intensities of the protein bands of each target were quantified in their respective fluorescent channels using Odyssey software (background subtraction top/bottom of quantified band). The integrated intensity for PMEL17 Mβ fragment was normalized to the integrated intensity for actin and displayed as % of control.

## Additional Information

**How to cite this article**: Shimshek, D. R. *et al.* Pharmacological BACE1 and BACE2 inhibition induces hair depigmentation by inhibiting PMEL17 processing in mice. *Sci. Rep.* **6**, 21917; doi: 10.1038/srep21917 (2016).

## Supplementary Material

Supplementary Information
