# Supplementary material for: Pharmacological BACE1 and BACE2 inhibition induces hair depigmentation by inhibiting PMEL17 processing in mice
Source: Sci Rep. 2016 Feb 25;6:21917. doi: 10.1038/srep21917 (PMC4766495; doi:10.1038/srep21917)
Supplement: Supplementary Information [file srep21917-s1.pdf]

Supplementary Figures and Figure Legends

Pharmacological BACE1 and BACE2 inhibition induces hair depigmentation by inhibiting PMEL17 processing in mice

Derya R. Shimshek[1+], Laura H. Jacobson[1*], Carine Kolly[2], Natasa Zamurovic[2], Kamal Kumar Balavenkatraman[2], Laurent Morawiec[2], Robert Kreutzer[2], Juliane Schelle[5], Mathias Jucker[5], Barbara Bertschi[2], Diethilde Theil[2], Annabelle Heier[2], Karine Bigot[2], Karen Beltz[3], Rainer Machauer[4], Irena Brzak[1], Ludovic Perrot[1] and Ulf Neumann[1]

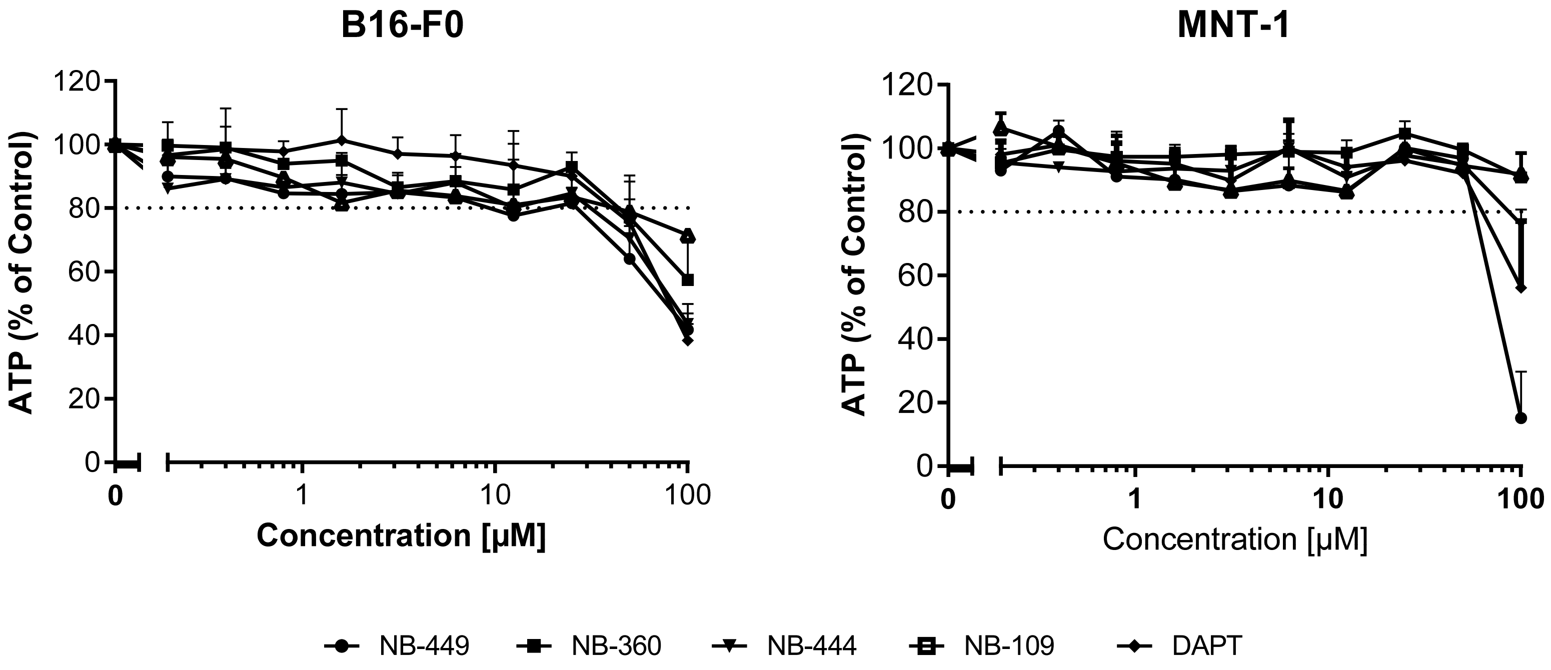

Supplementary Figure 1

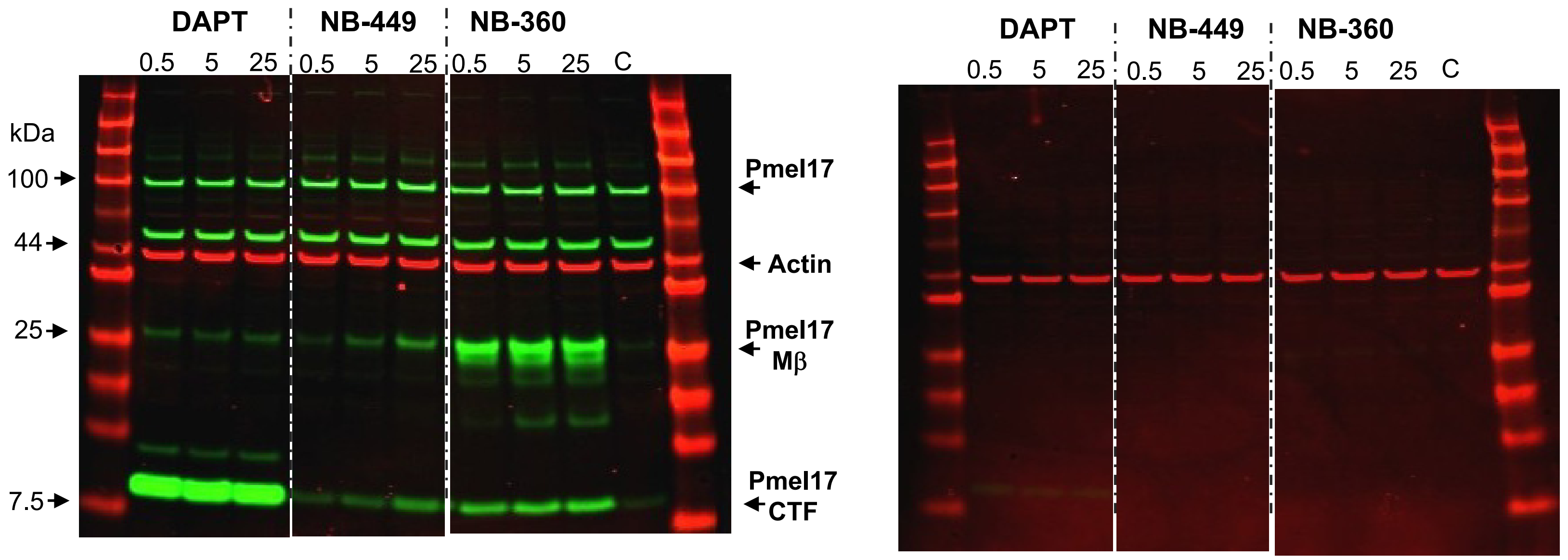

Supplementary Figure 2

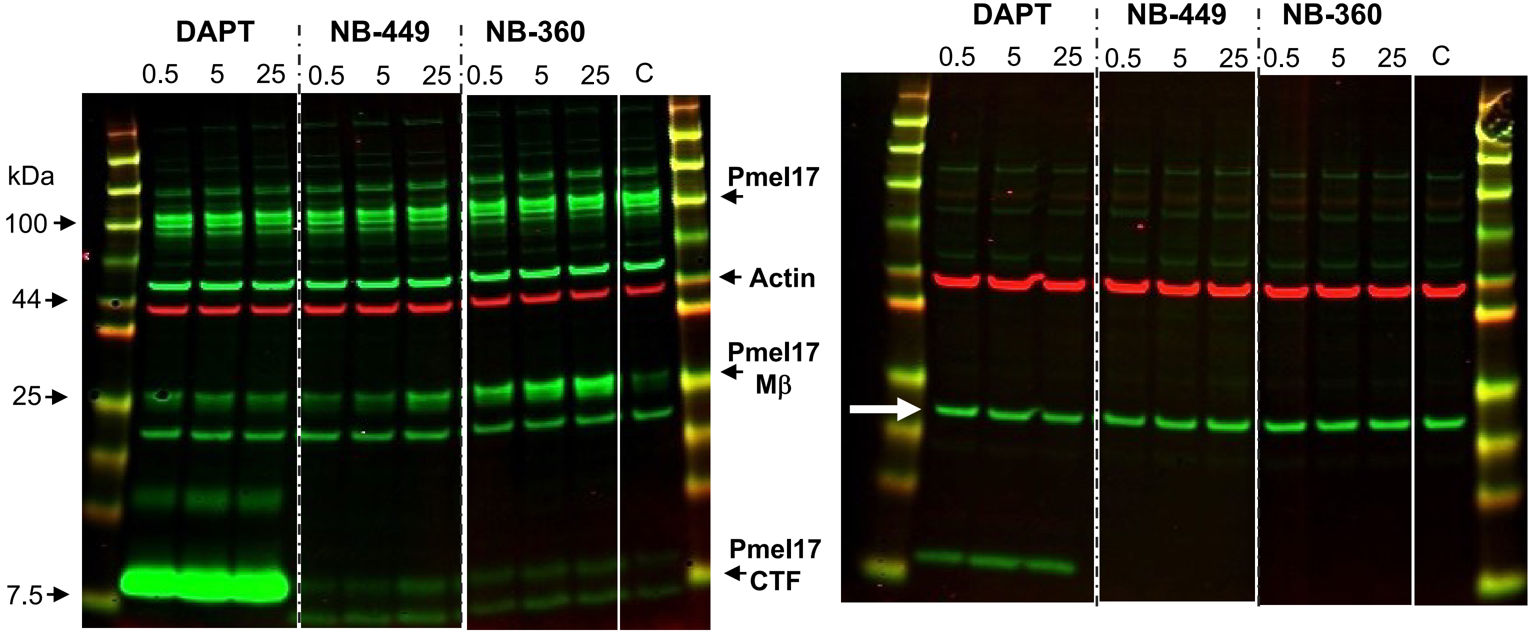

Supplementary Figure 3

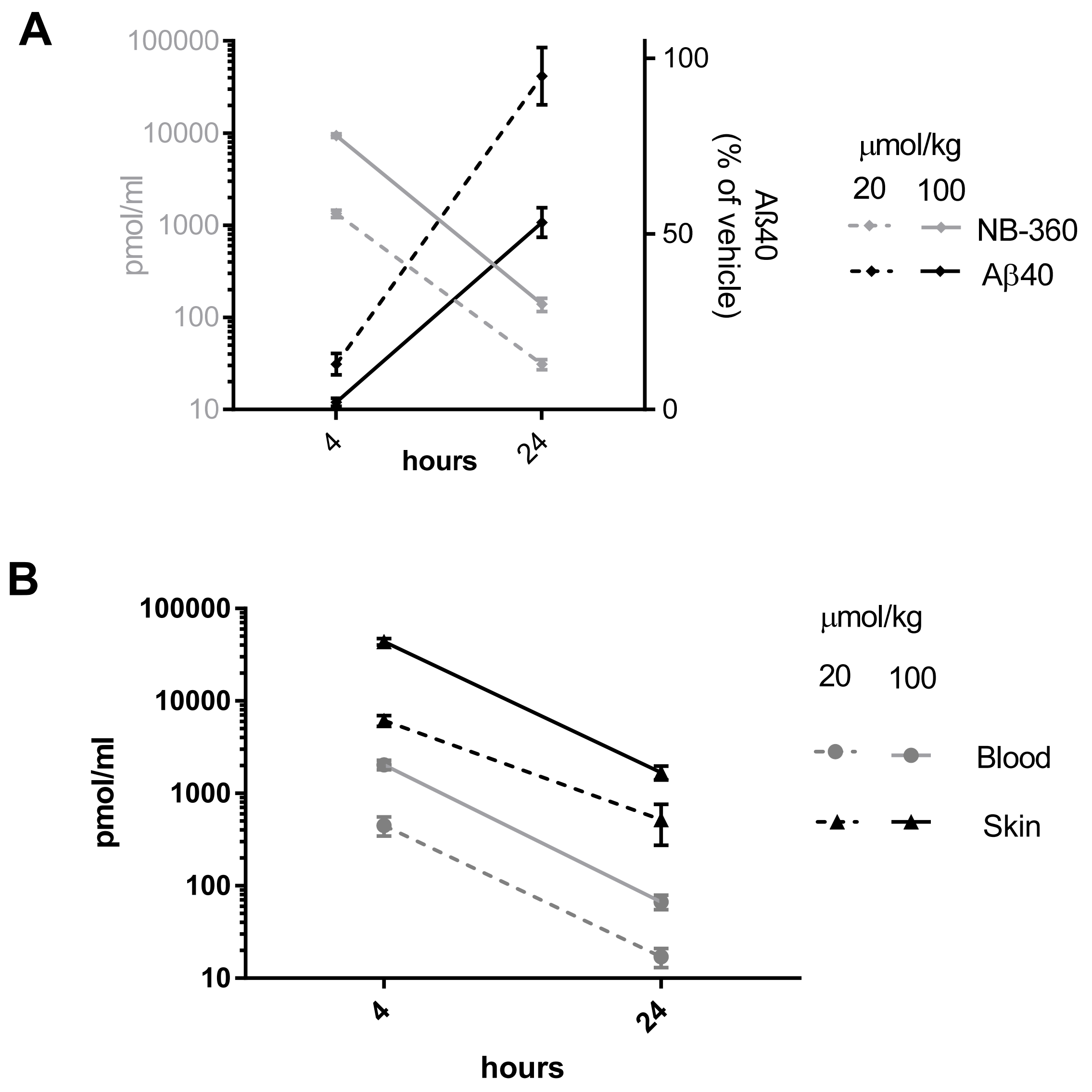

Supplementary Figure 4

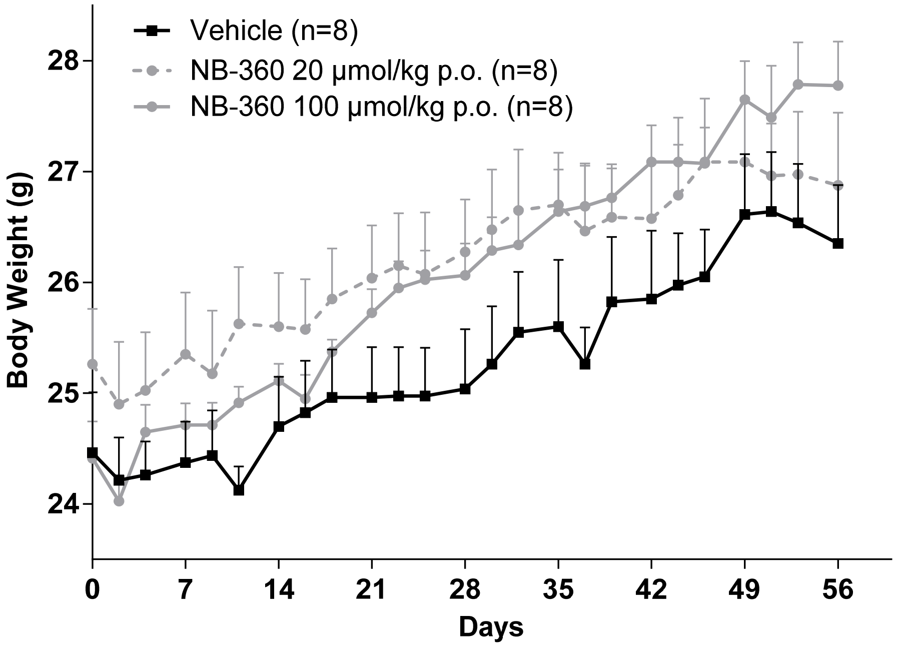

Supplementary Figure 5

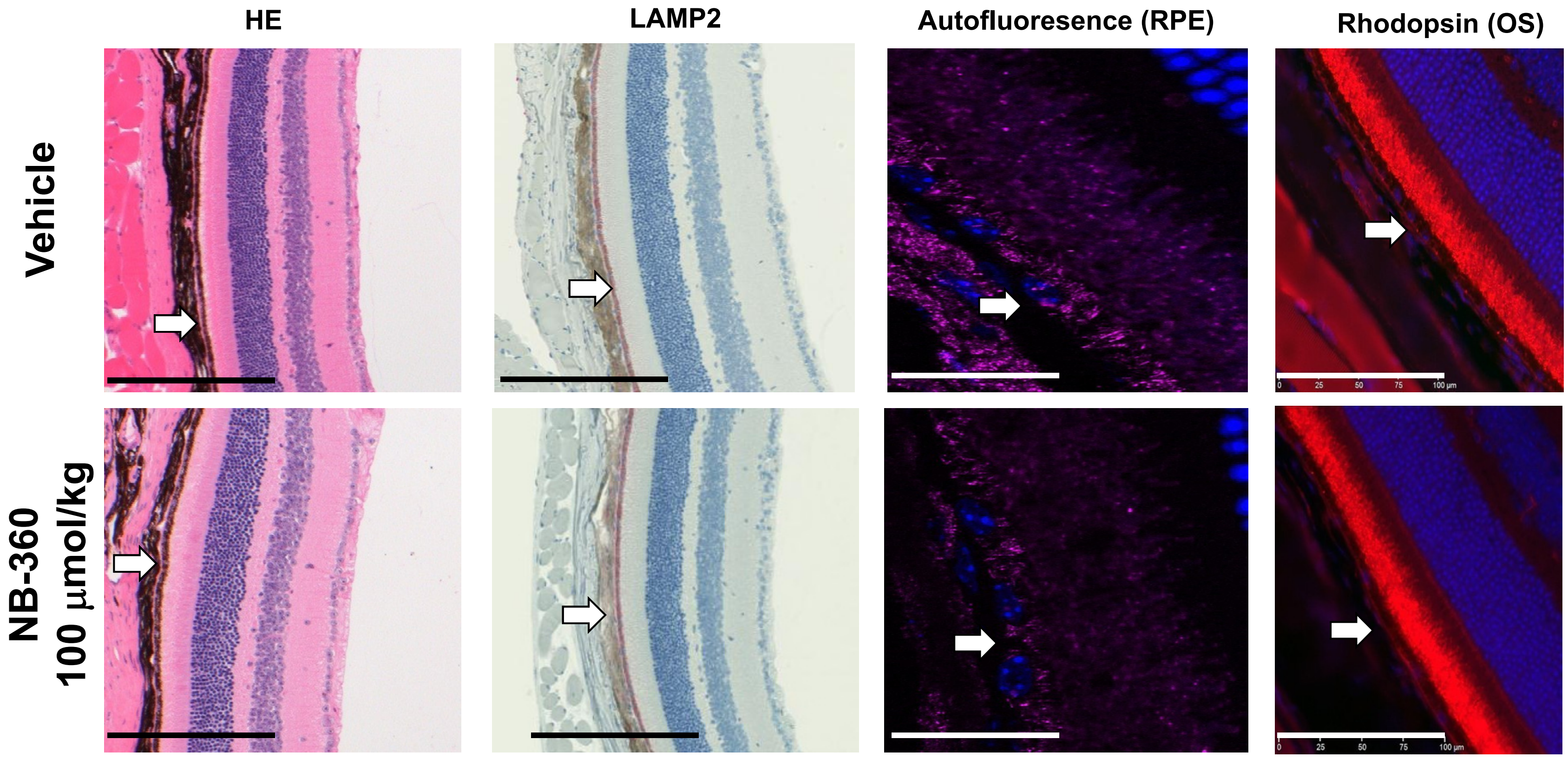

Supplementary Figure 6

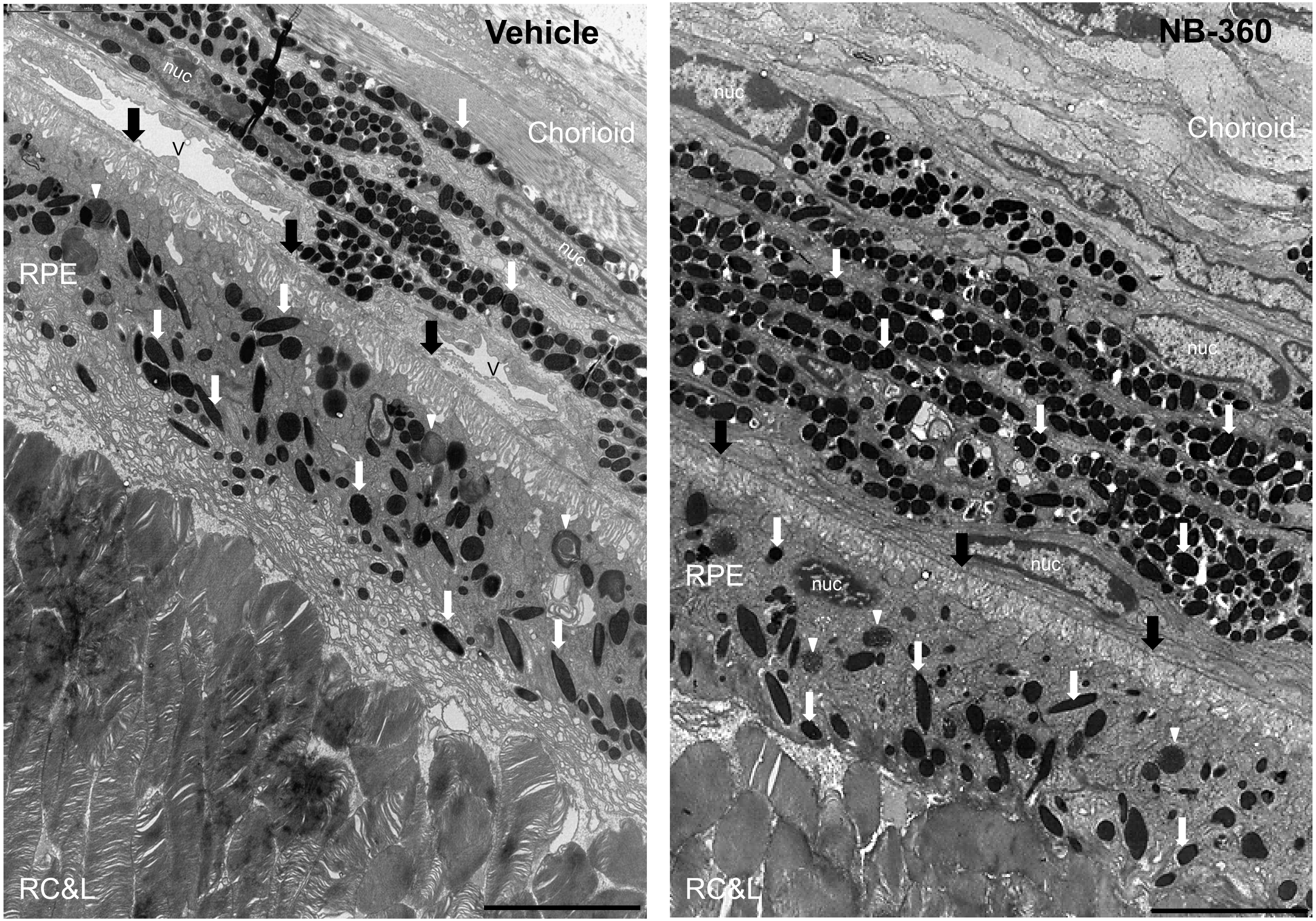

Supplementary Figure 7

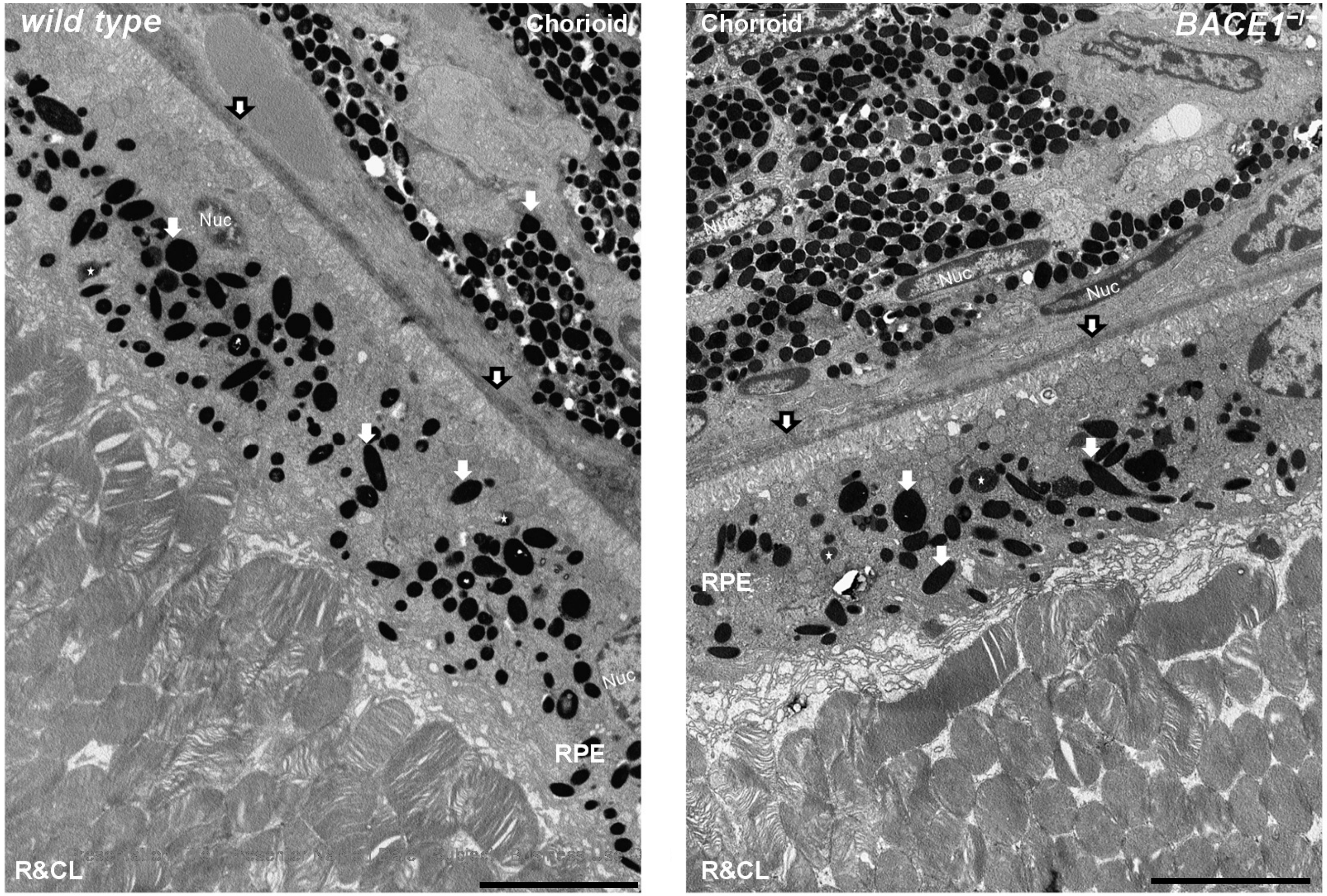

Supplementary Figure 8

Supplementary Figure 1. Intracellular ATP content of mouse (B16-F0) and human (MNT-1) melanocytes after 24 hours of treatment. Line graphs represent cellular ATP levels after treatment with increasing concentrations (0.2μM – 100μM) of the test compound compared to their untreated controls. Dotted line represents the non-cytotoxic threshold concentrations. Mean±SD.

Supplementary Figure 2. Evaluation of the specificity of the PMEL17 antibody in B16-F0 mouse melanocytes. Immunoblot detecting PMEL17 full length as well as the proteolytic cleavage products thereof, PMEL17 Mb and PMEL17 CTF (C-terminal fragment) as indicated. B16-F0 cells were treated with compound at different concentrations as indicated (in μM). Loading control: b-actin. On the left, immunoblot performed with the PMEL17 antibody (2μg/ml) and competed with immunogenic peptide (200-fold molar excess). No unspecific bands were observed.

Supplementary Figure 3. Evaluation of the specificity of the PMEL17 antibody in MNT-1 human melanocytes. Immunoblot detecting PMEL17 full length as well as the proteolytic cleavage products thereof, PMEL17 Mb and PMEL17 CTF (C-terminal fragment) as indicated. MNT-1 cells were treated with compound at different concentrations as indicated (in μM). Loading control: b-actin. On the left, immunoblot performed with the PMEL17 antibody (2μg/ml) and competed with immunogenic peptide (200-fold molar excess). One unspecific band could be observed (white arrow).

Supplementary Figure 4. 8-week NB-360 treatment effects on brain Abeta40 lowering and the tissue exposure of NB-360 in blood, skin and brain in male C57BL/6J mice. (**A**) Dose-dependent brain levels of NB-360 after 4 and 24 hours at the end of the 8-week chronic daily treatment at 20 and 100μmol/kg shown in Fig. 1A and their respective vehicle-normalized (in %) Abeta40 lowering effect in the mouse brain. (**B**) Dose-dependent blood and skin levels of NB-360 after 4

and 24 hours at the end of the 8-week chronic daily oral treatment at 20 and 100µmol/kg depicted in Fig. 1A.

Supplementary Figure 5. Body weight monitoring of male C57BL/6J mice treated for 8 weeks with NB-360 as depicted in Fig. 1A.

Supplementary Figure 6. 6-week NB-360 treated female wildtype C57BL/6 mice of the APP51 line with 100µmol/kg show no eye pathology compared to vehicle. Hematoxylin and Eosin (HE), LAMP2 for lysosomes, autofluorescence for lipofuscin accumulation and rhodopsin for the rod outer segment. Arrow: retinal pigmented epithelium (RPE). scale bars – 100µm.

Supplementary Figure 7. 6-week NB-360 treated female wildtype C57BL/6 mice of the APP51 line with 100µmol/kg showed no eye ultrastructural changes in the RPE and choroid compared to vehicle. White arrow: pigment granules; white arrowheads: phagolysosomes; black arrows: Bruch's membrane; nuc – nucleus; RC&L – rod and cones layer; scale bars – 20µm

Supplementary Figure 8. 6-month old *bace1*$^{-/-}$ showed no ultrastructural changes in the RPE and choroid compared to wildtype. White arrow: pigment granules; white stars: phagolysosomes; black arrows: Bruch's membrane; nuc: nucleus; RC&L: rod and cones layer; scale bars – 20µm
